# Risk of Diabetes Mellitus in Persons with and without HIV: A Danish Nationwide Population-Based Cohort Study

**DOI:** 10.1371/journal.pone.0044575

**Published:** 2012-09-12

**Authors:** Line D. Rasmussen, Elisabeth R. Mathiesen, Gitte Kronborg, Court Pedersen, Jan Gerstoft, Niels Obel

**Affiliations:** 1 Department of Infectious Diseases, Odense University Hospital, Odense, Denmark; 2 Department of Endocrinology, Copenhagen University Hospital, Rigshospitalet, Copenhagen, Denmark; 3 Department of Infectious Diseases, Copenhagen University Hospital, Hvidovre, Denmark; 4 Department of Infectious Diseases, Copenhagen University Hospital, Rigshospitalet, Copenhagen, Denmark; University of Buea, Cameroon

## Abstract

**Objective:**

In a nationwide, population-based cohort study we assessed the risk of diabetes mellitus (DM) in HIV-infected individuals compared with the general population, and evaluated the impact of risk factors for DM in HIV-infected individuals.

**Methods:**

We identified 4,984 Danish-born HIV-infected individuals from the Danish HIV Cohort Study and a Danish born population-based age- and gender-matched comparison cohort of 19,936 individuals (study period: 1996–2009). Data on DM was obtained from the Danish National Hospital Registry and the Danish National Prescription Registry. Incidence rate ratios (IRR) and impact of risk factors including exposure to Highly Active Antiretroviral Therapy (HAART) and antiretroviral drugs were estimated by Poisson regression analyses.

**Results:**

In the period 1996–1999 risk of DM was higher in HIV-infected individuals compared to the comparison cohort (adjusted IRR: 2.83; 95%CI: 1.57–5.09), both before (adjusted IRR: 2.40; 95%CI: 1.03–5.62) and after HAART initiation (adjusted IRR: 3.24; 95% CI: 1.42–7.39). In the period 1999–2010 the risk of DM in HIV-infected individuals did not differ from that of the comparison cohort (adjusted IRR: 0.90; 95% CI: 0.72–1.13), although the risk was decreased before HAART-initiation (adjusted IRR: 0.45; 95%CI: 0.21–0.96). Increasing age, BMI and the presence of lipoatrophy increased the risk of DM, as did exposure to indinavir, saquinavir, stavudine and didanosine.

**Conclusion:**

Native HIV–infected individuals do not have an increased risk of developing DM compared to a native background population after year 1998. Some antiretroviral drugs, not used in modern antiretroviral treatment, seem to increase the risk of DM.

## Introduction

Since the late nineties, studies on HIV-infected individuals have reported a wide spectrum of metabolic alterations associated with Highly Active Antiretroviral therapy (HAART) including changes in glucose homeostasis and fat redistribution [1]–[3]. As the lifespan of HIV-infected individuals have been prolonged, due to a decline in HIV-associated morbidity and mortality on account of HAART [4]–[5], such metabolic imbalances could affect the long-tem prognosis due to progression of insulin resistance to diabetes mellitus (DM) and subsequent risk of end-organ disease.

In addition to the well known risk factors for DM [6], immunodeficiency, lipodystrophy, socioeconomic class, concurrent hepatitis C infection (HCV), and drug abuse have been described as possible risk factors [3], [7]–[13]. Since the US Food and Drug Administration in 1997 issued a warning on the diabetogenic effects of protease inhibitors (PIs), risk of glucose alterations in HIV-infected individuals have been largely attributed to this drug class [14]–[18]. Additionally, nucleotide reverse transcriptase inhibitors (NRTIs) have been proposed to accelerate the pathogenetic mechanisms of DM development, but the data are limited [7], [9]–[11], [18]–[22]. As insulin resistance and impaired glucose tolerance induced by HAART might act as a precursor of DM, risk of DM might be increased in the HAART era. Several studies have addressed the risk of DM in the HIV-infected population [7], [9]–[11], [18]–[19], [21]–[24], but the results are conflicting and the majority of the studies are hampered by mixed ethnicity and lack of a comparison cohort from the general population.

We aimed to conduct a nationwide, population-based cohort study in the period 1 January 1996 to 1 January 2010 to investigate the risk of DM in HIV-infected individuals compared to that of the general population. To evaluate the impact of certain risk factors we further examined the influence of age, body mass index (BMI), lipoatrophy, HAART and specific antiretroviral drugs on risk of DM in HIV-infected individuals.

## Methods

### Setting

As of 1 January 2010 Denmark had a population of 5.5 million, with an estimated HIV prevalence of 0.1% among adults [25]–[26]. Treatment of HIV infection is restricted to eight specialized centers, where patients are seen on an outpatient basis at intended intervals of 12 weeks. Antiretroviral treatment is provided free-of-charge. During the follow-up period of the study, national criteria for initiating HAART were HIV-related disease, acute HIV infection, pregnancy, CD4 cell count <300 cells/µl, and, until 2001, plasma HIV-RNA >100,000 copies/ml. HAART was defined as a treatment regimen of at least three antiretroviral drugs or a treatment regimen including a combination of a non-nucleoside reverse transcriptase inhibitor and a boosted protease inhibitor and/or integrase inhibitor. Structured treatment interruptions have generally not been used in Denmark.

### Data Sources

We used the unique 10-digit civil registration number assigned to all individuals in Denmark at birth or upon immigration to link data from the following registers:

#### The Danish HIV Cohort Study (DHCS)

DHCS, which has been described in detail elsewhere [27], is a nationwide, prospective, population-based cohort study of all Danish HIV-infected individuals treated in one of the above mentioned centers since 1 January 1995. DHCS is still ongoing, thus consecutively enrolling new HIV-infected individuals and immigrants with HIV infection. As all HIV-infected individuals are referred to one of the above mentioned centers at diagnosis, and HAART is only available in these centers, DHCS includes almost all individuals diagnosed with HIV in Denmark.

#### The Danish Civil Registration System (DCRS)

DCRS, established in 1968, is a national registry which stores information on vital status, residency, and immigration/emigration for all Danish residents [28].

#### The Danish National Hospital Registry (DNHR)

DNHR, established in 1977, records data on all patients discharged from non-psychiatric hospitals in Denmark. Diagnoses are coded according to the *International Classification of Diseases* [8^th^ revision (ICD-8) until December 31 1993 and 10^th^ revision (ICD-10) thereafter] [29]. Since 1995 data on outpatients and emergency patients have been included.

#### The Danish National Prescription Registry (DNPR)

DNPR, established in1994 by the Danish Medicines Agency at the National Board of Health [30], records individual-level data on all redeemed prescriptions dispensed at Danish community pharmacies with complete data since 1 January 1995. The register includes variables related to the drug user, prescriber and pharmacy as well as the dispensing such as date of dispensing, product code and name, Anatomical Therapeutic Chemical Classification (ATC) code, dosage form and strength.

### Study Populations

#### HIV cohort

The HIV cohort consisted of all Danish born HIV-infected individuals, older than 16 years at HIV diagnosis, identified from DHCS. The index date was defined as the date of HIV diagnosis or January 1 1996 whichever was more recent. Individuals, with prevalent DM diagnoses at or a prescription of an anti-diabetic drug prior to index date, were excluded.

#### General population comparison cohort

The comparison cohort consisted of 4 age-and-gender matched population controls for each HIV-infected individual identified from DCRS. Only Danish born individuals were selected. Additional criteria for inclusion included being alive and living in Denmark on index date, having no registration of DM in DNHR prior to index date and having no redeemed prescription of an anti-diabetic drug prior to index date. Index date for the comparison cohort was defined as the index date of the corresponding HIV-infected individual.

### Outcome

We identified the first date an individual was registered with a DM code in DNHR or the date of first redemption of a prescription of an anti-diabetic drug following index date and defined this as new-onset DM (ICD8 and ICD-10 diagnosis codes: 249.00–259.09, E10.0-E14.9; ATC codes for insulin and analogues, and oral anti-diabetic drugs: A10AB01-A10AE05, A10BA02-A10BX07– codes and descriptions are further provided in the Appendix S1 and S2). As the ICD8/ICD10 codes registered in DNHR defines DM as either insulin dependent DM (IDDM) or non-insulin-dependent (NIDDM), it is not possible to distinguish between type 1 and type 2 DM based on this information. We therefore defined Type 2 DM as individuals assigned a NIDDM code (E11/259), individuals assigned one of the three less specific DM forms (E12, E13, E14), or individuals who had ever redeemed a prescription of an oral anti-diabetic drug. This approach is in agreement with the approach used by Petersen et al [31]. We furthermore defined type 2DM as individuals who had redeemed prescriptions on insulin products but with no prior IDDM code (E10/249) or individuals with an IDDM code who had never initiated insulin therapy. Type 1 DM was defined as individuals with an IDDM code who initiated insulin therapy and did not fulfil any of the above mentioned criteria.

### Confounding Variables

In Denmark the racial distribution in the HIV-infected population is different from that of the general population. As we had no access to racial data for the comparison cohort, we restricted the analyses to native (Danish born) individuals for both groups (HIV-infected individuals vs. comparison cohort). In the final model we included the following covariates to control for potential confounding: - Age (fitted as a time-updated variable- categorized in 9 age intervals, split at the ages 30, 35, 40, 45, 50, 55, 60, 65), gender and calendar year (categorized in 5 calendar intervals, split at 1 January 1999, 2001, 2004 and 2007). Analysis on HIV-infected individuals were further adjusted according to the presence of HCV and/or intravenous drug abuse (IDU) (IDU if registered as route of infection in DHCS and HCV defined as being seropositive for HCV and/or having a positive HCV RNA registered in DHCS - Hepatitis testing is performed routinely at the first visit). Data on height and weight at index date was extracted from DHCS and BMI was calculated and grouped as defined in the Appendix S3. Lipoatrophy was extracted from DHCS as the first date an HIV-infected individual presented with lipoatrophy and introduced as a time-up dated variable to evaluate the risk of DM prior to and after this date.

### Statistical Analysis

Time was computed from index date until date of new-onset DM, date of death, emigration, lost to follow up or 1 January 2010, whichever occurred first. Cumulative incidence function was used to illustrate time to first occurrence of DM recognizing death as a competing risk. We used Poisson regression analysis to compute incidence rates (IR) and incidence rate ratios (IRR), as a measure of the relative risk, and 95% confidence intervals (CI), comparing the risk of DM in HIV-infected individuals with that of the comparison cohort. In a robustness analysis we restricted the outcome of the analyses to type 2 DM as defined in the above section, however, as this did not change the estimates substantially all further analysis were performed with DM as endpoint. The analyses were adjusted for potential confounding factors as described in the above section. Due to effect modification by calendar time, analysis comparing HIV-infected individuals and the comparison cohort individuals were analysed separately according to split in calendar time (time-updated variable). In the HIV-infected individuals risk of DM was assessed according to BMI level, age and lipoatrophy. To evaluate the impact of HAART on risk of DM we included date of HAART initiation as a time-updated variable and assessed the IRR of DM in the non-HAART period and the HAART period for the HIV-infected individuals compared to the comparison cohort individuals.

To estimate the impact of antiretroviral drugs (indinavir, saquinavir, nelfinavir, atazanavir, lopinavir +/−ritonavir, PIs in general, and stavudine, zidovudine, didanosine, abacavir, tenofovir and lamivudine) on the risk of DM, we performed analyses in which only HIV-infected individuals initiating HAART were included. Time was calculated from date of HAART initiation. The first initiation of the specific drug was handled as a time-updated variable and first date of a CD4 cell count >200 cells/µl after start of HAART was included for confounder control. In these analyses an individual who initiated a specific antiretroviral drug was considered on this drug for the rest of the observation period independent of cessation or changes in antiretroviral therapy. Additionally, risk of DM was assessed according to the cumulative use of HAART.

Statistical analyses were performed using SPSS version 19.0 (SPSS Inc., Chicago, Illinois, USA), STATA software, version 11.0 (Stata Corporation, college Station Texas, USA) and R version 2.11.1. Data from DNHR and DNPR was obtained with approval from the Danish Registry Board. The study was approved by the Danish Data Protection Agency (jr. no 2008–41–1781).

## Results

We studied a total of 3,540 Danish born HIV-infected individuals and 14,160 Danish born comparison cohort individuals. Characteristics are summarized in table 1.

**Table 1:** Basic characteristics for Danish born HIV-infected individuals and comparison cohort individuals.

	Danish born	
	HIV-infected individuals	Comparison cohort individuals
	(N = 3,540)	(N = 14,160)
**Age at index date, median years (IQR)**	38.7 (32.2–46.6)	38.7 (32.2–46.6)
**Male gender, N (%)**	2,977 (84.1)	11,908 (84.1)
**Caucasian race, N (%)**	3,451 (97.5)	–
**Body mass index (BMI) at index date:**		
**Underweight (BMI: <18.5), N (%)**	199 (5.6)	–
**Normal weight (BMI: 18.5–24.9), N (%)**	1,651 (46.6)	–
**Overweight (BMI: 25.0–29.9), N (%)**	539 (15.2)	–
**Obesity (BMI: >30.0), N (%)**	104 (2.9)	–
**Missing data on BMI, N (%)**	1,047 (29.6)	–
**Infection mode:**		–
**MSM, N (%)**	1,881 (53.1)	–
**Heterosexual contact, N (%)**	1,009 (28.3)	–
**Intravenous drug abuse, N (%)**	453 (12.8)	–
**Other/unknown, N (%)**	197 (6.6)	–
**Hepatitis C co-infection or intravenous drug abuse, N (%)**	677 (19.1)	–
**No of patients diagnosed with HIV-infection prior to 1996, N (%)**	1,674 (44.5)	–
**No of patients with AIDS diagnosed prior to index date, N (%)**	367 (10.4)	–
**CD4 cell count at index date, median cells/µL (IQR)**	300 (120–497)	–
**Duration of follow-up, PYR**	28,342	136,365.5
**Duration of follow-up, median-years (IQR)**	8.0 (3.4–14.0)	11.2 (5.5–14.0)
**Death during follow-up, N (%)**	845 (23.9)	643 (4.5)
**Emigration during follow-up, N (%)**	67 (1.9)	140 (1.0)
**Lost to follow-up, N (%)**	2 (0.1)	2 (0.0)
**New-onset diabetes mellitus (DM) after index date, N (%)**	105 (3.0)	528 (3.7)
**Type 1 DM, N (%)**	10 (0.3)	17 (0.1)
**Type 2 DM, N (%)**	95 (2.7)	511 (3.6)
**Age at DM diagnosis, median years (IQR)**	52.6 (43.5–60.6)	54.0 (46.6–61.0)
**Lipoatrophy prior to DM diagnosis, N (%)**	21 (20.0)	–
**Initiated HAART prior to DM diagnosis, N (%)**	90 (85.7)	–
**CD4 cell count at DM diagnosis, median** **cells/µL (IQR)**	437 (199–655)	–
**IR of diabetes mellitus per 1,000 PYR (95% CI)**	3.70 (3.06–4.49)	3.87 (3.56–4.22)

**Abbreviations:** IQR: Interquartile range; IR: Incidence Rate; 95% CI: 95% Confidence Interval; PYR: Person-years; MSM: men who have sex with men; DM: Diabetes Mellitus; HAART: Highly Active Antiretroviral Therapy.

Overall DM was diagnosed in 105 (3.0%) HIV-infected individuals and in 528 (3.7%) comparison cohort individuals (Table 1).

**Figure 1:**
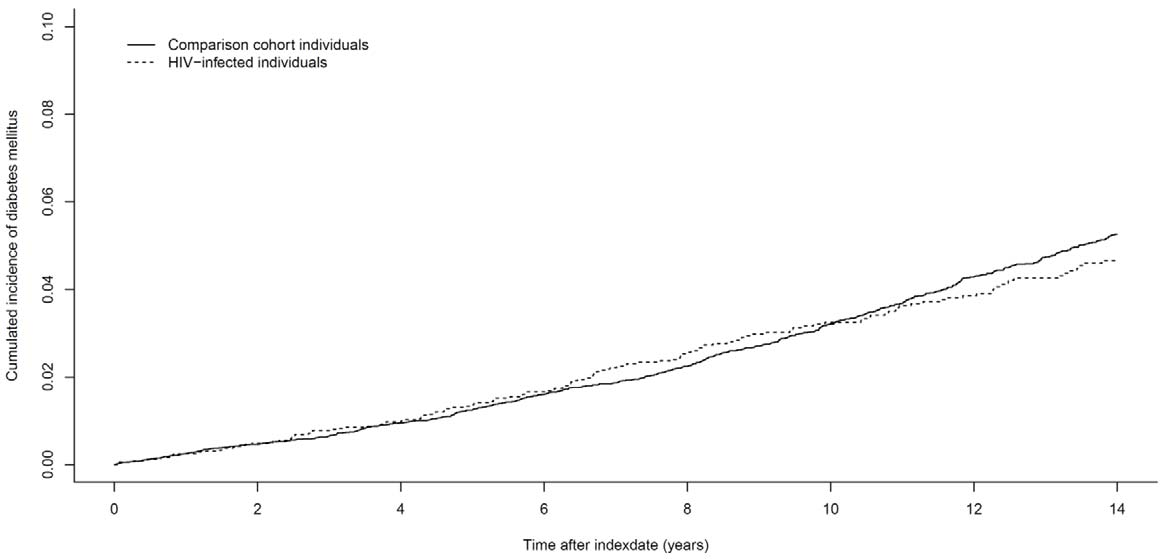
Cumulative incidence of diabetes mellitus in Danish born HIV-infected individuals compared to a Danish born comparison cohort. (Index date: the latest of 1 January 1996 or date of HIV diagnosis).

Figure 1 presents the cumulative incidence curve for time from index date to DM for HIV-infected individuals and comparison cohort individuals.

In Danish born HIV-infected individuals risk of DM did not differ from that of a Danish born comparison cohort (adjusted IRR: 1.02; 95% CI: 0.83–1.26). As we found an interaction with calendar time, indicating a significant difference in the association between HIV and DM in the different calendar years, we performed the analyses separately according to this time-updated variable. This phenomenon is not visible in figure 1 as the x-axis presents time from index date (i.e. HIV diagnosis) and not calendar time. In the years 1996–1998 the risk of DM was significantly higher in HIV-infected individuals relative to the comparison cohort (adjusted IRR: 2.83; 95%CI: 1.57–5.09), but thereafter the risk was not increased (adjusted IRR: 0.90; 95%CI: 0.72–1.13) (table 2). Before 1999 we observed a higher risk of DM prior to (adjusted IRR: 2.40; 95%CI: 1.03–5.62) and after HAART-initiation (adjusted IRR: 3.24; 95%CI: 1.42–7.39) in the HIV-infected individuals than the comparison cohort. In contrast the risk of DM in the period 1999–2010 showed a statistically significant lower risk in the non-HAART period (adjusted IRR: 0.45; 95%CI: 0.21–0.96) and no increased risk after initiation of HAART (adjusted IRR: 1.00; 95%CI: 0.79–1.28) (Table 2).

**Table 2:** Risk of diabetes mellitus (DM) in Danish born HIV-infected individuals compared to Danish born comparison cohort individuals.

	DM events/PYR for HIV-infected individuals	DM events/PYR for comparison cohort individuals	IRR of diabetes mellitus in Danish born HIV-infected individuals versus comparison cohort individuals	
			Unadjusted IRR (95% CI)	Adjusted* IRR (95% CI)
**Diabetes mellitus:**	105/28,342	528/136,366	0.96 (0.78–1.18)	1.02 (0.83–1.26)
**Stratified on gender:**				
**Males:**	90/23,308	467/112,690	0.93 (0.74–1.17)	0.99 (0.79–1.25)
**Females:**	15/5,034	61/23,675	1.16 (0.66–2.03)	1.22 (0.69–2.15)
**Analysed according to split in calendar time (time-updated variable)**			**Unadjusted IRR (95% CI)**	**Adjusted** ** **IRR (95% CI)**
**01.01.1996–31.12.1998**	18/4,768	29/20,992	2.73 (1.52–4.92)	2.83 (1.57–5.09)
**01.01.1999–01.01.2010**	87/23,574	499/115,374	0.85 (0.68–1.07)	0.90 (0.72–1.13)
**Analysed according to HAART initiation and split in calendar time (Time-updated variables)**			**Unadjusted IRR (95% CI)**	**Adjusted** ** **IRR (95% CI)**
**01.01.1996–31.12.1998**				
**NON-HAART**	8/2,904	16/13,223	2.28 (0.97–5.32)	2.40 (1.03–5.62)
**HAART**	10/1,864	13/7,768	3.21 (1.41–7.31)	3.24 (1.42–7.39)
**01.01.1999–01.01.2010**				
**Non-HAART**	7/5,028	122/30,221	0.34 (0.16–0.74)	0.45 (0.21–0.96)
**HAART**	80/18,546	377/85,153	0.97 (0.77–1.24)	1.00 (0.79–1.28)

**Abbreviations:** IRR: Incidence rate ratio; 95% CI: Confidence Interval; PYR: Person-years; HAART: Highly Active Antiretroviral Therapy.

* Adjusted for age (split at 30, 35, 40, 45, 50, 55, 60 and 65), gender and calendar time (split at 3, 5, 8 and 11 years after January 1, 1996).

** Analysed separately according to split in calendar time (split at 3 years after January 1, 1996) and adjusted for age (split at 30, 35, 40, 45, 50, 55, 60, and 65) and gender. For time periods after 31 December1998 the analysis were further adjusted for calendar time (split at 5, 8, 11 years after January 1, 1996).

HIV–infected individuals showed an increasing risk of DM with increasing age and BMI (Table 3). Also a diagnosis of lipoatrophy increased the risk substantially (adjusted IRR: 2.30; 95%CI: 1.39–3.80). We found no association between the cumulative effect of HAART and risk of diabetes (results not shown).

**Table 3:** Impact of body mass index (BMI), age and lipoatrophy on risk of diabetes mellitus in Danish born HIV-infected individuals.

			IRR of Diabetes Mellitus in Danish born HIV-infected individuals	
	DM events (105)	PYR (28,342)	Unadjusted IRR (95% CI)	Adjusted* IRR (95% CI)
**Baseline variables:**				
**Body mass index (BMI):**				
**Underweight (BMI: <18.5)**	3	1,513	0.85 (0.26–2.77)	0.89 (0.27–2.90)
**Normal weight (BMI: 18.5–24.9)**	36	15,466	Ref (1)	Ref (1)
**Overweight (BMI: 25.0–29.9)**	22	5,239	1. 80 (1.06–3.07)	1.60 (0.94–2.73)
**Obesity (BMI: >30.0)**	21	899	10.04 (5.86–17.20)	9.25 (5.37–15.94)
**Time-updated variables:**				
**Age periods (years):**				
**0–29**	2	1,776	Ref (1)	Ref (1)
**30–39**	14	8.576	1.45 (0.33–6.38)	1.10 (0.25–4.85)
**40–49**	30	9,821	2.71 (0.65–11.35)	2.01 (0.48–8.47)
**50–59**	32	5,821	4.88 (1.17–20.37)	3.73 (0.89–15.66)
**60+**	27	2,348	10.21 (2.43–42.95)	8.16 (1.91–34.74)
**Lipoatrophy:**				
**No lipoatrophy**	84	26,182	Ref (1)	Ref (1)
**Lipoatrophy**	21	2,160	3.03 (1.88–4.89)	2.30 (1.39–3.80)

**Abbreviations:** IRR: Incidence rate ratio; 95% CI: Confidence Interval; PYR: Person-years;

* Adjusted for age (split at 30, 35, 40, 45, 50, 55, 60, and 65), gender and calendar time (split at 3, 5, 8 and 11 years after January 1, 1996) and Hepatitis C infection or Intravenous drug abuse.

Initiation of stavudine (adjusted IRR: 1.81; 95%CI:1.19–2.75) and saquinavir (adjusted IRR: 1.53; 95%CI: 1.01–2.34) significantly increased the risk of DM compared with the HAART-period prior to initiation of these antiretroviral drugs, while a trend towards a higher incidence of DM in patients on indinavir (adjusted IRR: 1.38; 95%CI: 0.91–2.11) and didanosine (adjusted IRR: 1.52; 95%CI: 0.99–2.34) was found. Nelfinavir, atazanavir, ritonavir +/−lopinavir and PI in general, as well as zidovudine, abacavir, tenofovir and lamivudine did not increase the risk of DM (Table 4).

The performed robustness analysis, in which only cases defined as type 2 DM as previously defined was used as outcome, showed no major changes in the estimates of the relative risk (results not shown).

**Table 4:** Impact of specific antiretroviral drugs on risk of diabetes mellitus (DM) in Danish born HIV-infected individuals on HAART.

			IRR of diabetes mellitus in Danish born HIV-infected individuals on HAART	
Time-updated variables:	DM events (90)	PYR (20,410)	Unadjusted IRR (95% CI)	Adjusted* IRR (95% CI)
**PROTEASE INHIBITORS:**				
	19	4,172	Ref (1)	Ref (1)
**Ever exposed to PI**	71	16,238	0.96 (0.58–1.59)	0.95 (0.56–1.62)
	46	12,182	Ref (1)	Ref (1)
**Ever exposed to indinavir**	44	8,228	1.42 (0.94–2.14)	1.38 (0.91–2.11)
	51	13,556	Ref (1)	Ref (1)
**Ever exposed to saquinavir**	39	6,854	1.51 (1.00–2.29)	1.53 (1.01–2.34)
	70	15,464	Ref (1)	Ref (1)
**Ever exposed to nelfinavir**	20	4,946	0.89 (0.54–1.47)	0.95 (0.57–1.57)
	79	18,127	Ref (1)	Ref (1)
**Ever exposed to atazanavir**	11	2,283	1.11 (0.59–2.08)	0.95 (0.49–1.88)
	35	8,477	Ref (1)	Ref (1)
**Ever exposed to ritonavir**	55	11,932	1.12 (0.73–1.71)	1.11 (0.72–1.70)
	76	17,168	Ref (1)	Ref (1)
**Ever exposed to lopinavir/ritonavir**	14	3,242	0.98 (0.55–1.73)	0.94 (0.52–1.69)
**NUCLEOSIDE REVERSE TRANSCRIPTASE INHIBITORS:**				
	46	13,561	Ref (1)	Ref (1)
**Ever exposed to stavudine**	44	6,849	1.89 (1.25–2.86)	1.81 (1.19–2.75)
	8	2,112	Ref (1)	Ref (1)
**Ever exposed to zidovudine**	82	18,298	1.18 (0.57–2.44)	1.10 (0.53–2.30)
	56	14,863	Ref (1)	Ref (1)
**Ever exposed to dianosine**	34	5,547	1.63 (1.06–2.49)	1.52 (0.99–2.34)
	48	11,831	Ref (1)	Ref (1)
**Ever exposed to abacavir**	42	8,579	1.21 (0.80–1.83)	1.09 (0.68–1.73)
	73	16,876	Ref (1)	Ref (1)
**Ever exposed to tenofovir**	17	3,534	1.11 (0.66–1.89)	1.06 (0.59–1.91)
	4	1,113	Ref (1)	Ref (1)
**Ever exposed to lamivudine**	86	19,297	1.24 (0.46–3.38)	1.15 (0.42–3.17)

**Abbreviations:** IRR: Incidence rate ratio; 95% CI: Confidence Interval; PYR: Person-years; Ref: Reference group ∼ HIV-infected individuals on HAART not exposed to the investigated antiretroviral drug.

* Adjusted for age (split at 30, 40, 50 and 60), gender, calendar time (split at 3, 5, 8 and 11 years after January 1, 1996), Hepatitis C infection or intravenous drug abuse, and CD4 cell count (split at date of CD4 cell count >200 cells/µL after start of HAART).

## Discussion

In the period 1999–2010 we observed no increased risk of DM in HIV-infected individuals compared to the general population. However, in this period the risk was decreased prior to HAART initiation. In the years 1996–1998 the risk was increased irrespective of HAART treatment. Increasing age, BMI and the presence of lipoatrophy was associated with an increased risk of DM as was exposure to indinavir, saquinavir, stavudine and didanosine.

The strengths of our study include access to nationwide population-based cohorts with long and complete follow-up, which also enabled us to identify a population-based, age and gender matched comparison cohort. We obtained data on study endpoints from the same data sources, which minimised differential misclassification. As non-Caucasians have higher rates of DM [32] the risk posed by HIV and HAART may be overestimated in studies primarily including non-Caucasian individuals. To minimise confounding posed by differences in ethnicity between groups (HIV-infected/comparison cohort), we restricted the analysis to native individuals. We furthermore adjusted for potential confounding factors and evaluated clinical relevant effect modifications. We are not aware of other studies with a similar design.

Due to the study design we had no access to fasting glucose measurements and had to rely on hospital registry-based discharge diagnoses, diagnosis from outpatient clinics and data on redeemed prescriptions of anti-diabetic drugs. As non-fasting glucose concentration is measured at least once yearly in Danish HIV-clinics, HIV-infected individuals might be prone to an earlier DM diagnosis than the general population. However, without using regular fasting glucose measurements for both groups, the true DM incidence could be underestimated, and the categorisation of the individual as type 2 DM might be delayed for several years. As life style modifications are first line treatment for type 2 DM, individuals in our study may remain unregistered in this period, which makes a window for withdrawal and switch of antiretroviral drugs with the risk of reversed causality problems [7]. Furthermore, as we used an intent-to-continue-treatment approach the associations seen were related to individuals who had ever been exposed to the drugs rather than to those who are currently exposed. For drugs that might have been used for shorter periods in the earlier years, this could bias the results. We used DM in general as our primary endpoint as type 1 and type 2 DM could not be unambiguous discriminated in the registries [31], [33]. A similar approach has been used by others [31] and a robustness analyses demonstrated that our results were not severely biased by this design.

As lipoatrophy was defined as fat redistribution and registered in DHCS without standardized assessments such as DEXA, MRI or CT scans, lipoatrophy could be a weak predictor of true lipodystrophy. Additionally, BMI was estimated at index date. As the weight can change dramatically over years, especially in the HIV-infected population, we are aware that the absence of the weight dynamic could have affected the results; however, using time-dependent measures for BMI did not modify the findings in a recent French study [22]. Finally, as we had no data on socioeconomic status, level of exercise, family history of DM or information on BMI for the comparison cohort, we could not adjust for these potential confounders.

Despite the extensive literature on metabolic alterations in HIV-infected individuals, no consensus regarding risk of DM has been reached. In a recent French study (APROCO-COPILOTE/1997–1999), Capeau et al. [22] found a markedly higher incidence of DM (14.1 per 1,000PYR) than we did and what was reported for the HIV-uninfected French population, the European general population and other HIV-infected populations (4–6/1,000 PYR) [22]. However, measures of incidence might vary due to differences in factors such as age, gender, race and BMI. In comparison a study from the MACS cohort [19] (1999–2003) found that HIV-infected men on HAART compared to HIV-negative men had a more than 4 times increased risk of developing DM when adjusting for age and BMI, whereas the incidence of DM in the HIV-infected individuals not on HAART did not differ substantially from that of HIV-negative individuals. These findings contrast our results from the years subsequent to 31 December 1998 as well as the gender specific results from the WIHS (2001–2003 and 2002–2004), MS and CHAMPS cohort (2000–2005) [21], [23], [34], that found no statistically significant impact of HIV or HAART on risk of DM. In addition, the VACS cohort (2000–2005) [8] even found that HIV was associated with a statistically significant lower prevalence of DM (adjusted odds ratio: 0.84; 95% CI 0.72–0.97).

In the years 1996–1998 we found a 2.83 times higher risk of DM in the HIV-infected population than in the comparison cohort. As the increased risk was observed both in the period before and in the period after HAART initiation, the effect seems not only associated with the use of toxic antiretroviral drug-regimens. This early period reflects a population considerably affected by the HIV infection and therefore differs from the later period. But, drugs with potentially diabetogenic effects such as Pentamidin Isothionate, used for the prevention and treatment of Pneumocystis Jirovici, and mono-therapy with first generation antiretroviral drugs like stavudine and indinavir, were also used in the pre-HAART era. In addition, it must be emphasized that the observed increased risk of DM in 1996–1998 may rely on a small number of events. In the study by Capeau et al. [22] incidence peaked in 1999–2000 and gradually decreased thereafter. This was partly explained by exposure to high levels of first generation antiretroviral drugs and to lipodystrophy. Similarly, De Wit et al. (the D:A:D-study) [9] also found that earlier calendar years (1999–2000) was associated with a higher risk of DM. However, contrary to the ongoing design of our study, in which patients were consecutively recruited during the entire study period, these studies consisted of closed cohorts [9], [22].

After 1998 we found that HIV-infected individuals not on HAART had a decreased risk of DM which is in accordance with the non-significant trend found by Tien et al. [21]. A large Danish population-based survey (Inter99, 1999–2000) showed a higher proportion of overweight (39.5%) and obesity (16.3%) in the general population (age 30–60 years) than in our HIV-infected cohort [6]. This potentially lower BMI in our HIV-infected cohort compared to the general population could have lead to an underestimation of the impact of HIV infection per se. We therefore presume that the decreased risk of DM in the non-HAART period could be due to lower weight of the untreated individuals and less focus on DM before start of HAART; however, an effect related purely to small numbers cannot be excluded.

Due to a rising prevalence of overweight and obesity in developed countries, the prevalence of DM is increasing rapidly [6], [35]. Our findings of an increased risk of DM with increasing age and BMI indicate that traditional risk factors seem to apply in the same way in HIV-infected individuals as for the background population. We therefore suggest that HIV patients are screened for DM in accordance with local general guidelines.

Several studies have found an increased risk of DM in HIV-infected individuals on HAART compared to HAART-naïve individuals [7]–[8], [18]–[19], [24]. In contrast, studies of the cumulative effect of HAART as well as individual drug classes have shown conflicting results [7], [9]–[10], [18]–[23], [34]. We observed an increased risk of DM in HIV-infected individuals treated with HAART when compared to HIV-infected individuals not on HAART. However, this effect does not stem from a higher risk of DM in HAART treated patients compared to the general population, but relies on a lower incidence of DM in the non-HAART period. We saw an increased risk of DM in the early calendar period which is in accordance with the use of indinavir, saquinavir, stavudine and didanosine in the early HAART era. This observation is in accordance with the results by Capeau et al. [22].

In some studies treatment with PIs in general has been found associated with higher risk of DM [10], [18]. This contrasts our observations of no increased DM risk. The discrepancies might rely on marked variation in risk of DM between different PIs as the effects of individual drugs might be diluted when analysing PI as a class. Prior studies have indicated a direct blockage of GLUT-4 induced by indinavir [36]–[37] and thus an acute onset and possible reversible effect after drug discontinuation. In addition, Ledergerber et al. [7] examined 6,513 HIV-infected individuals from the Swiss Cohort Study and found a higher risk of DM in association with indinavir, which is consistent with the trend found in our study and the results of others [11], [20], [22].

Some NRTIs have been proposed to be associated with a higher risk of DM [7], [9], [11], [19], [22]. Ledergerber et al. [7] found a strong association between current therapy with NRTI, and risk of incident DM. Stavudine has consistently been associated with a higher risk of lipoatrophy, hyperinsulinemia and DM [9], [11], [19]–[20], [22]. Consistent with our results the APROCO-COPILOTE-study and the D:A:D-study [9], [22] found an increased risk of DM in association with stavudine and didanosine as well as a significant association with lipodystrophy. However, adjusting for lipodystrophy did not modify the relationship substantially, which might indicate an effect of NRTIs independent of lipodystrophy [9].

In conclusion, native HIV-infected individuals have no increased risk of DM compared to native individuals from the background population after year 1998. Some antiretroviral drugs that are only used infrequently in modern antiretroviral treatment seem to increase the risk of DM.

## Supporting Information

Appendix S1 **Diagnostic codes of diabetes mellitus (ICD 8 and ICD 10 codes).**
(DOC)

Appendix S2 **ATC codes of antidiabetic drugs (Insulin and analogues, and oral antidiabetic drugs).**
(DOC)

Appendix S3 **Definition and grouping of body mass index.**
(DOC)
