# Supplementary material for: Risk of Diabetes Mellitus in Persons with and without HIV: A Danish Nationwide Population-Based Cohort Study
Source: PLoS One. 2012 Sep 12;7(9):e44575. doi: 10.1371/journal.pone.0044575 (PMC3440341; doi:10.1371/journal.pone.0044575)
Supplement: Appendix S1 — Diagnostic codes of diabetes mellitus (ICD 8 and ICD 10 codes). (DOC) [file pone.0044575.s001.doc]

**APPENDIX S1**

**DIAGNOSTIC CODES OF DIABETES MELLITUS:**

**ICD 10:**

E10.0-10.9: Insulin dependent diabetes mellitus (IDDM)

E11.0-11.9: Non-insulin dependent diabetes mellitus (NIDDM)

E12.0-12.9: Diabetes mellitus (DM) due to malnutrition

E13.0-13.9: Other DM

E14.0-14.9: unspecified DM

**ICD8:**

249.00-249.09: IDDM

259.00-259.09: NIDDM
