# Supplementary material for: Risk of Diabetes Mellitus in Persons with and without HIV: A Danish Nationwide Population-Based Cohort Study
Source: PLoS One. 2012 Sep 12;7(9):e44575. doi: 10.1371/journal.pone.0044575 (PMC3440341; doi:10.1371/journal.pone.0044575)
Supplement: Appendix S2 — ATC codes of antidiabetic drugs (Insulin and analogues, and oral antidiabetic drugs). (DOC) [file pone.0044575.s002.doc]

**APPENDIX S2**

**ANTIDIABETIC DRUGS USED SINCE 1995 (INCLUDING ATC CHANGES):**

(ATC=Anatomical Therapeutic Chemical Classification)

**INSULIN AND ANALOGUES:**

A10AB01 Actrapid

A10AB01 Actrapid Novolet

A10AB01 Actrapid Penfil

  A10AB01 [Humulin Regular](http://pro.medicin.dk/Medicin/Praeparater/1584)

  A10AB01 [Insuman Rapid Solostar](http://pro.medicin.dk/Medicin/Praeparater/5952)

A10AB01 Velosulin

  A10AB04 Humalog

A10AB04 Humalog Pen

  A10AB05 NovoRapid

  A10AB05 NovoRapid FlexPen

  A10AB05 NovoRapid Penfill

  A10AB06 Apidra

  A10AB06 Apidra OptiSet

  A10AB06 Apidra Solo Star

  A10AC01 [Humulin NPH](http://pro.medicin.dk/Medicin/Praeparater/1585)

  A10AC01 [Humulin NPH](http://pro.medicin.dk/Medicin/Praeparater/1585) Pen

  A10AC01 Insulatard

  A10AC01 Insulatard FlexPen

  A10AC01 Insulatard InnoLet

  A10AC01 Insulatard NovoLet

  A10AC01 Insulatard Penfil

A10AC01 Insuman Basal

A10AC01 Monotard

A10AD01 Humulin Mix

A10AD01 Insuman Comb 25

A10AD01 Mixtard 10 InnoLet

A10AD01 Mixtard 10 Penfil

A10AD01 Mixtard 20 NovoLet

A10AD01 Mixtard 20 Penfil

A10AD01 Mixtard 20/80 Penfil

A10AD01 Mixtard 30

A10AD01 Mixtard 30 InnoLet

A10AD01 Mixtard 30 NovoLet

A10AD01 Mixtard 30 Penfil

A10AD01 Mixtard 30/70 Pen

A10AD01 Mixtard 40 NovoLet

A10AD01 Mixtard 40 Penfil

A10AD01 Mixtard 40/60 Pen

A10AD01 Mixtard 50 NovoLet

A10AD01 Mixtard 50 Penfil

A10AD01 Mixtard 50/50 Pen

  A10AD04 [Humalog Mix25](http://pro.medicin.dk/Medicin/Praeparater/6411) KwikPen

  A10AD04 [Humalog Mix25 100 E/ml Pen](http://pro.medicin.dk/Medicin/Praeparater/2507)

  A10AD04 [Humalog Mix50 100 E/ml Pen](http://pro.medicin.dk/Medicin/Praeparater/2652)

  A10AD05 NovoMix 30 FlexPen

  A10AD05 NovoMix 30 Penfil

  A10AD05 NovoMix 50 FlexPen

  A10AD05 NovoMix 70 FlexPen

  A10AE04 Lantus

  A10AE04 Lantus Optiset

  A10AE04 Lantus Solo Star

  A10AE05 Levemir

- The ATC-codes A10AA01-04 (Insulin and analogues – quick effect, long efficiency, combination therapy and long efficiency) were changed 1 January 1997 to the ATC-codes A10AB, A10AC, A10AD, A10AE as described above.

**ORAL ANTIDIABETIC DRUGS**

A10BA02 Glucophage

A10BA02 Metformin “1A Farma”

A10BA02 [Metformin "Actavis"](http://pro.medicin.dk/Medicin/Praeparater/2900)

A10BA02 [Metformin "Actavis"](http://pro.medicin.dk/Medicin/Praeparater/2900)

  A10BA02 [Metformin "Aurobindo"](http://pro.medicin.dk/Medicin/Praeparater/4844)

A10BA02 [Metformin "Biochemise"](http://pro.medicin.dk/Medicin/Praeparater/2900)

  A10BA02 [Metformin "Bluefish"](http://pro.medicin.dk/Medicin/Praeparater/4769)

A10BA02 [Metformin "Hexal"](http://pro.medicin.dk/Medicin/Praeparater/2900)

  A10BA02 [Metformin "Mylan"](http://pro.medicin.dk/Medicin/Praeparater/4881)

  A10BA02 [Metformin "Orifarm"](http://pro.medicin.dk/Medicin/Praeparater/5957)

  A10BA02 [Metformin "Sandoz"](http://pro.medicin.dk/Medicin/Praeparater/6396)

  A10BA02 [Metformin "Stada"](http://pro.medicin.dk/Medicin/Praeparater/3775)

  A10BA02 [Metformin "Teva"](http://pro.medicin.dk/Medicin/Praeparater/4219)

  A10BA02 [Orabet](http://pro.medicin.dk/Medicin/Praeparater/134)

A10BB01 Daonil

  A10BB01 [Hexaglucon](http://pro.medicin.dk/Medicin/Praeparater/1021)

  A10BB03 Arcosal

A10BB03 Tolbutamid “DAK”

A10BB07 Glibenese

  A10BB07 Mindiab

  A10BB09 [Diamicron Uno](http://pro.medicin.dk/Medicin/Praeparater/125)

A10BB09 Gliclzid ”Alternova”

  A10BB09 [Gliclazid "KRKA"](http://pro.medicin.dk/Medicin/Praeparater/4437)

  A10BB09 [Gliclazid "ratiopharm"](http://pro.medicin.dk/Medicin/Praeparater/4648)

  A10BB09 [Gliclazid "Stada"](http://pro.medicin.dk/Medicin/Praeparater/6140)

  A10BB09 [Gliclazid "Teva"](http://pro.medicin.dk/Medicin/Praeparater/4457)

A10BB09 Uni Diamicron

A10BB09 Unidiamicron

  A10BB12 Amaryl

A10BB12 Glimepirid “1A Farma”

  A10BB12 [Glimepirid "Actavis"](http://pro.medicin.dk/Medicin/Praeparater/3816)

  A10BB12 [Glimepirid "Alpharma"](http://pro.medicin.dk/Medicin/Praeparater/3816)

  A10BB12 [Glimepirid "Alternova"](http://pro.medicin.dk/Medicin/Praeparater/3816)

  A10BB12 [Glimepirid "BMM Pharma"](http://pro.medicin.dk/Medicin/Praeparater/4829)

  A10BB12 [Glimepirid "Copyfarm"](http://pro.medicin.dk/Medicin/Praeparater/3816)

  A10BB12 [Glimepirid "Hexal"](http://pro.medicin.dk/Medicin/Praeparater/3816)

  A10BB12 [Glimepirid "KRKA"](http://pro.medicin.dk/Medicin/Praeparater/3818)

  A10BB12 [Glimepirid "Mylan"](http://pro.medicin.dk/Medicin/Praeparater/3816)

  A10BB12 [Glimepirid "ratiopharm"](http://pro.medicin.dk/Medicin/Praeparater/3837)

  A10BB12 [Glimepirid "Sandoz"](http://pro.medicin.dk/Medicin/Praeparater/3815)

  A10BB12 [Glimepirid "Stada"](http://pro.medicin.dk/Medicin/Praeparater/3855)

  A10BB12 [Glimepirid "Teva"](http://pro.medicin.dk/Medicin/Praeparater/4304)

  A10BB12 [Glimepirid "Winthrop"](http://pro.medicin.dk/Medicin/Praeparater/3816)

A10BD03 Avandamet

A10BD04 Avaglim

  A10BD07 Janumet

  A10BD08 Eucreas

  A10BF01 Glucobay

A10BG02 Avandia

  A10BG03 Actos

  A10BH01 Januvia

  A10BH02 Galvus

  A10BH03 Onglyza

  A10BX02 NovoNorm

  A10BX04 [Byetta](http://pro.medicin.dk/Medicin/Praeparater/4153)

  A10BX07 Victoza
