# Supplementary material for: Risk of Diabetes Mellitus in Persons with and without HIV: A Danish Nationwide Population-Based Cohort Study
Source: PLoS One. 2012 Sep 12;7(9):e44575. doi: 10.1371/journal.pone.0044575 (PMC3440341; doi:10.1371/journal.pone.0044575)
Supplement: Appendix S3 — Definition and grouping of body mass index. (DOC) [file pone.0044575.s003.doc]

**APPENDIX S3**

**DEFINITION OF BODY MASS INDEX (BMI):**

BMI was defined as weight in kilograms divided by height in meters squared (BMI= kg/(m)^2^).

**BMI grouping:**

Underweight: BMI: < 18.5

Normal weight: BMI: 18.5-24.9

Overweight: BMI: 18.5-24.9

Obesity: BMI: > 30.0
